# Titanium-Mediated Organic Electrosynthesis

**DOI:** 10.1021/acscatal.5c03780

**Published:** 2025-07-21

**Authors:** Julius Kuzmin, Cristiana Margarita, Guillermo Ahumada, Mainak Mitra, Helena Lundberg

**Affiliations:** † Department of Chemistry, KTH Royal Institute of Technology, Teknikringen 30, S-100 44 Stockholm, Sweden; ‡ Department of Chemistry, Burdwan Raj College, Purba Bardhaman, West Bengal 713104, India

**Keywords:** titanium, organic electrosynthesis, electrocatalysis, mediated electrolysis, reduction

## Abstract

Titanium is an earth abundant metal with low toxicity that is able to form complexes that mediate a wide range of organic transformations in both polar and radical manifolds. In context of the latter, the use of Ti-catalysts in electrosynthesis is surprisingly underexplored, considering the great potential for electrochemical (re)­generation of low-valent and catalytically active species. To spur further innovation in the field, this Review provides an overview of the current literature and discusses the limitations and possibilities for electrochemically driven Ti-catalysis.

## Graphical abstract

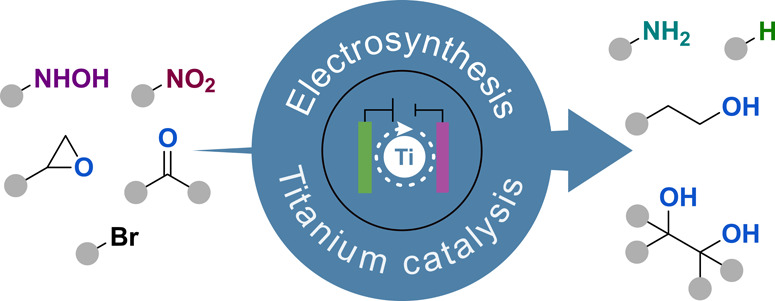

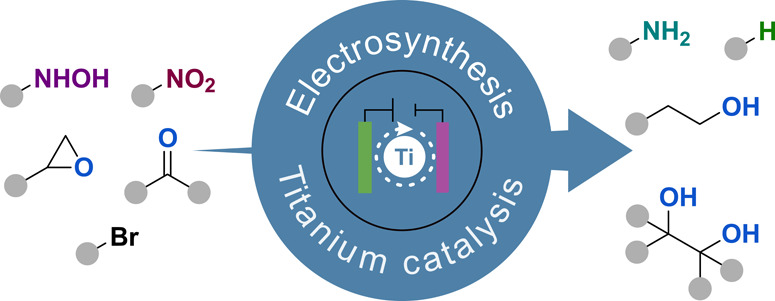

## Introduction

Titanium is the ninth most abundant element in Earth’s crust,^1,2^ with a considerably lower price and toxicity compared to many other transition metals, such as rhodium, palladium and platinum.^3,4^ These features make Ti complexes attractive as reagents and catalysts in organic synthesis (Scheme 1). Titanium readily forms Lewis acidic reagents and catalysts in its tetravalent d^0^ state. For example, tetravalent Ti-complexes have been successfully used for a wide range of transformations, including Sharpless epoxidation,^5^ the Pauson–Khand reaction,^6^ methylenation reactions with Tebbe or Petasis reagents,^7,8^ and olefin polymerization.^9^ Complexes based on titanium in lower valency states are also well-known in organic synthesis. The Nugent–RajanBabu complex—Cp_2_TiCl—is arguably the most well-explored species of the trivalent Ti-complexes and has successfully been used in both stoichiometric and catalytic amounts for a wide variety of transformations, including cyclizations and reductive couplings of, *e*.*g*., nitriles, carbonyls, imines or activated alkenes.^10–41^ Recently, the use of such Ti-complexes as redox reagents and/or catalysts for challenging deoxygenative transformations of alcohols has been reported.^42–48^ In addition, Ti-complexes of even lower oxidation states are of high synthetic utility, as evident from, e.g., McMurry couplings,^49^ N_2_ activation,^50–57^ desulfurative reactions,^58–62^ and reductive couplings.^63,64^

**Scheme 1:**
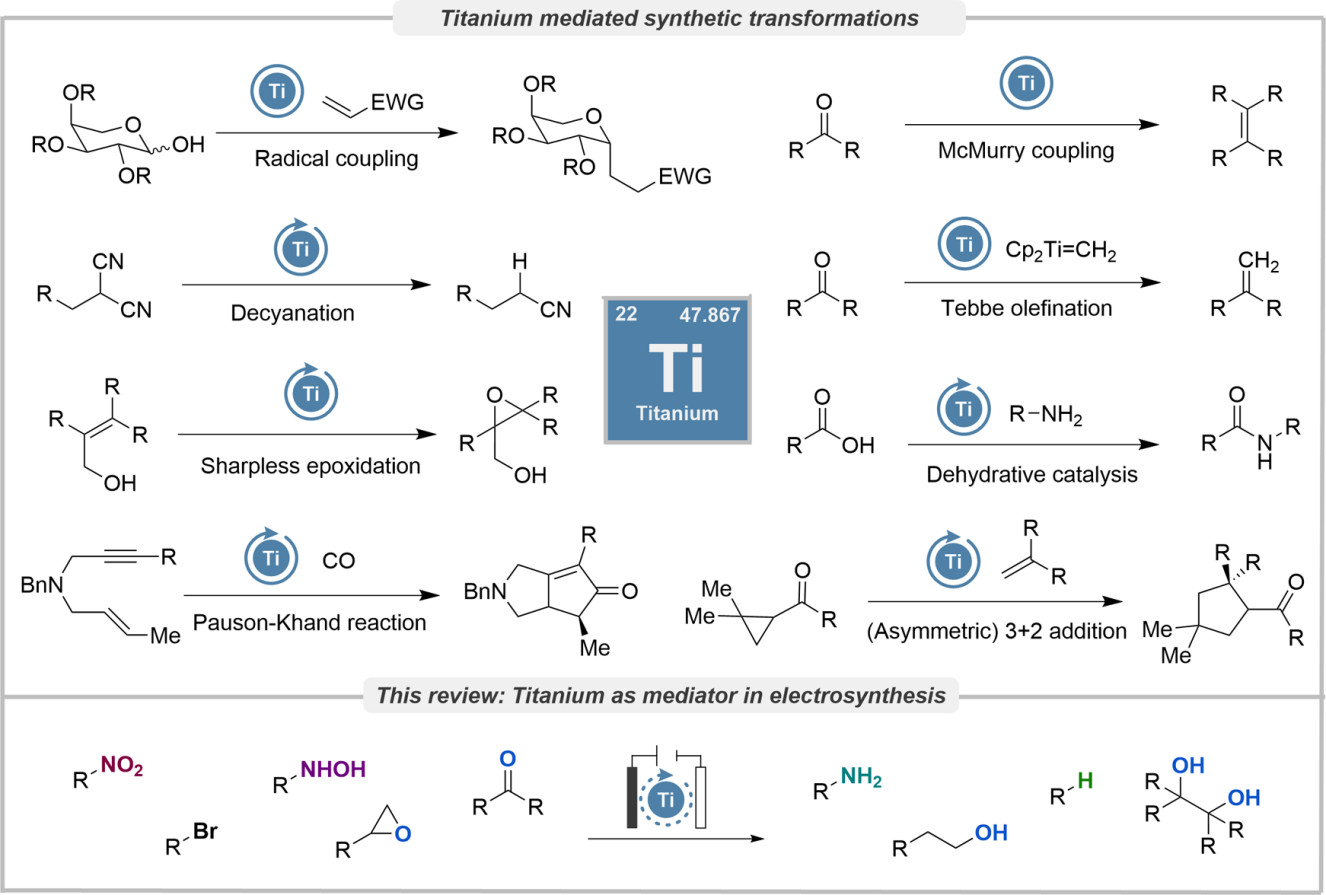
Examples of Ti-mediated organic transformations

Low-valent Ti-complexes are commonly formed from their tetravalent halide precursors with stoichiometric reductants,^65–69^ commonly Zn, Al, and Mn.^55,70–78^ This reduction strategy is well-established to regenerate the active catalyst in, e.g., reductive cross-electrophile couplings with or without catalysts based on other early transition metals such as Ni.^79–90^ While effective, this approach results in stoichiometric amounts of metal waste and, for this reason, electrosynthesis has emerged as an attractive alternative to drive catalytic turnover in net reductive processes.^91–93,81,94–97^ In addition, electrosynthesis may offer more precise control over redox processes through modulation of the applied potential, which can enhance the selectivity of synthetic transformations.^98–107^ In the context of low-valent titanium complexes, their use as reagents and catalysts under chemical and photochemical conditions has been rapidly increasing in recent years and the topic has been extensively reviewed.^11,29,30,108–111^ In contrast, the electrochemical (re)­generation of low-valent Ti-complexes for synthetic applications has been considerably less reported and remains relatively underutilized in both academic and industrial settings. This Review provides an overview of electrochemically driven Ti-mediated synthetic protocols, aiming to spur further innovation in this field of yet untapped potential and unlock new paradigms in Ti-catalysis for advancing sustainable organic synthesis. For consistency, potentials are reported versus the saturated calomel electrode (SCE). When other reference electrodes were used in the original studies, the reported values were converted using established conversion constants.^112^

## N–O Bond Cleavage

Electroreduction of oxidized nitrogen-containing functional groups such as hydroxylamines, nitro- and nitroso-groups produces versatile and reactive intermediates (Scheme 2A) that can be converted into high-value products.^113^ Redox mediators and catalysts have often been used to improve the Faradaic efficiency of the processes by preventing electrode passivation that may result upon direct electroreduction due to product adsorption to the electrode surface.^114,115^ In the context of titanium, complexes thereof have been utilized to facilitate N_2_ activation for the production of NH_3_,^116–126^ as well as for modification of organic compounds. For the conversion of nitro-groups to amines in the presence of Ti-complexes, acidic aqueous or two-phase systems under electrochemical conditions have been commonly reported.^127–136^ Supposedly, the acidic media was chosen to provide conductivity while preventing deactivation of the titanium catalysts due to their propensity for hydrolysis and precipitation to insoluble Ti­(OH)_3_ or TiO_2_ in neutral or basic aqueous solutions.^137,138^

**Scheme 2:**
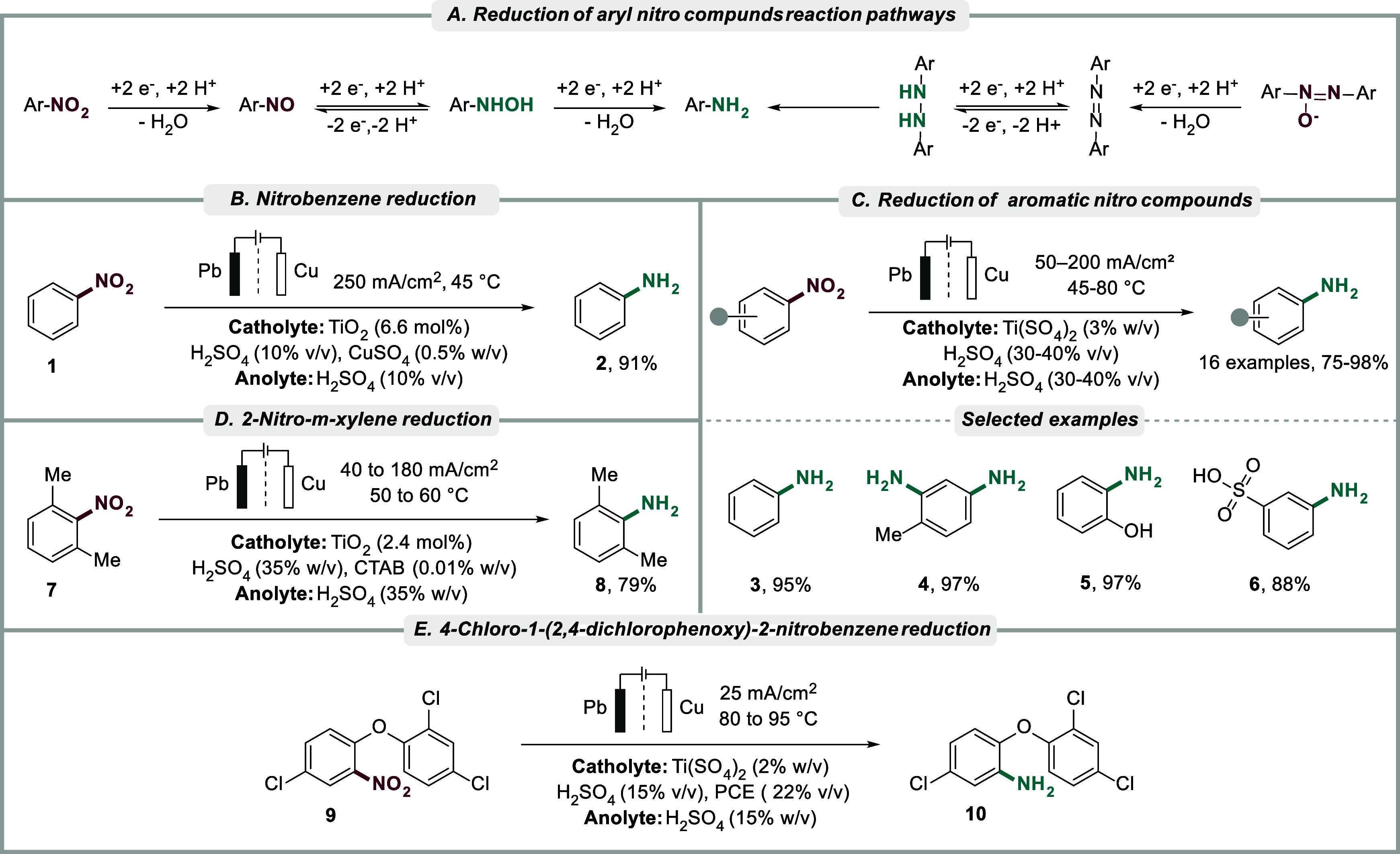
(A) Reaction pathways for reduction of aryl nitro compounds, (B) Reduction of nitrobenzene, (C) Reduction of various aromatic nitro compounds with selected scope, (D) Reduction of 2-nitro-*m*-xylene, and (E) Synthesis of antibacterial agent

Udupa et al. demonstrated that aniline **2** could be produced in yields up to 91% in acidic media (H_2_SO_4_) using a copper cathode and a lead anode in a divided cell (Scheme 2B).^139^ It was hypothesized that Ti­(III) forms from a tetravalent TiO_2_ precursor (used in 6.6 mol %) by cathodic reduction and acts as a chemical reductant for nitrobenzene, thereby reoxidizing to Ti­(IV). The authors demonstrated that the reduction of nitroarenes to the corresponding aniline is favored at low current densities, whereas high current densities favor the formation of an intermediate phenylhydroxylamine that is rapidly transformed into *p-*aminophenol. Under similar conditions, the same group demonstrated that 2-nitro-*m*-xylene **7** could be converted to 2-amino-*m*-xylene **8** in 79% yield and current efficiency up to 73% in the presence of 2.4% Ti­(SO_4_)_2_ (Scheme 2D).^140^ In this case, cetrimonium bromide (CTAB) was used both as an electrolyte and an emulsifying agent to enhance the solubility of the aromatic substrate. Interestingly, the addition of CTAB induced a shift in reduction potentials for the substrate and the Ti-catalyst. In the case of 2-nitro-*m*-xylene, the reduction potential was shifted to a more negative values, whereas the reduction of the Ti­(IV) species shifted to a more positive value. The mechanistic rationale behind these observations was not elaborated on. The authors presented an optimized process for the reduction of nitroaromatics utilizing a copper cathode and a lead anode in a divided cell with a porous pot diaphragm with 30–40% H_2_SO_4_ as the electrolyte with 2–3% Ti­(SO_4_)_2_ added to the catholyte (Scheme 2C).^128^ Using a similar approach, Thirunavukkarasu developed an electrochemical method using titanous sulfate (2.4% w/v) for the synthesis of triclosan **10**, a synthetic antibacterial agent commonly utilized in cosmetic formulations, obtaining a current efficiency of 70% (Scheme 2E).^141^

Mikhal’chenko and co-workers studied the electrochemical reduction of 1-ethyl-3-cyano-4-nitropyrazole to the corresponding amines in the presence of low-valent mediators based on Ti, V and Sn in a divided cell in aqueous acidic electrolyte (Scheme 3A).^136^ Interestingly, it was found that aminopyrazole **12** was selectively formed (56% yield) at reaction temperatures below 10 °C with TiCl_3_ as mediator, whereas the chlorinated analogue **13** was observed at higher temperature (60 °C, 73% yield). A mechanistically oriented study for the reduction of dinitrobenzenes indicated that TiCl_3_ may react at significantly higher rates compared to SnCl_2_ and VCl_2_, resulting in increased selectivity and yields of the corresponding diaminobenzene products.^142^ While SnCl_2_ too was found to be effective as mediator, the authors found that it resulted in more challenging isolation of the products due to the complexation of the latter with the metal ions. However, the opposite trend was observed for the electrochemical reduction of N-(2-nitro-4-R-phenyl)­pyridinium salts **14** into the corresponding anilines and subsequent intramolecular cyclization to furnish pyrido­[1,2-*a*]­benzimidazoles **15** in acidic aqueous-alcoholic media (Scheme 3B).^143^ In this case, the use of a Ti-based mediator resulted in lower yields of the pyridobenzimidazole compared to the use of a V-based mediator (52% and 64% yield, respectively), whereas the use of a Sn-mediator resulted in yields up to 92% with a 5-fold reduction in the reaction time compared to direct electroreduction in the absence of the mediator.

**Scheme 3:**
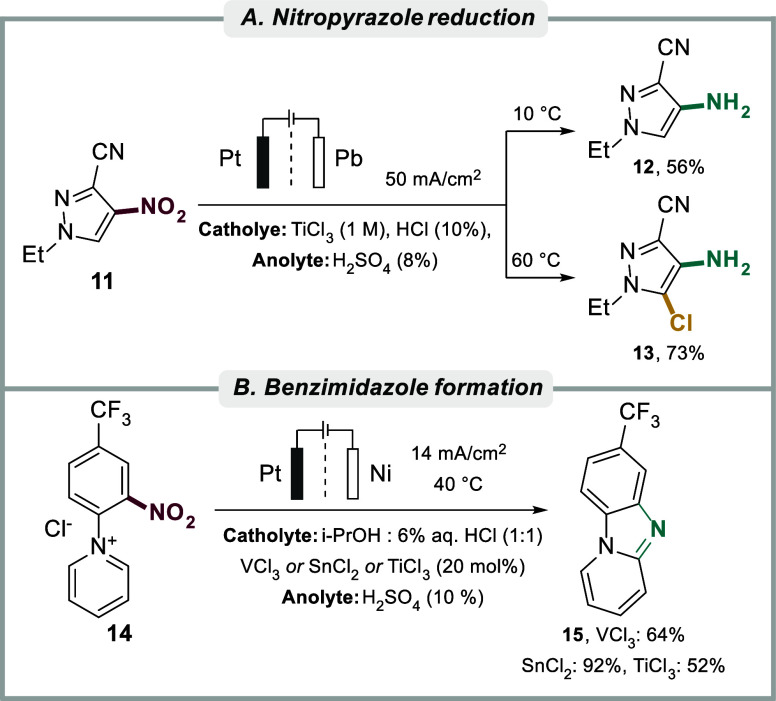
(A) Nitropyrazole reduction to the corresponding amine and (B) Benzimidazole formation from a nitrophenylpyridinium salt

Mousset et al. studied the cathodic reduction of 4,4′-dinitrodibenzyl under a variety of conditions.^144–146^ With titanium oxysulfate (TiOSO_4_) as a redox mediator, reduction of 4,4′-dinitrodibenzyl **16** proceeded smoothly via the dihydroxylamine intermediate **17**, to furnish the corresponding diamine product **19**. This compound was formed in 93% yield using a mercury cathode and a lead anode at a controlled potential of −0.60 V vs. SCE, in 1:1 H_2_SO_4_ (5N): EtOH solution at 60 °C (Scheme 4A). In contrast, the unmediated reaction resulted in a lower yield (75%) due to side-product formation.^144^ In subsequent work, Mousset and co-workers reported on the same transformation in a DMF electrolyte via electrochemical generation of a Ti­(III) mediator from Cp_2_TiCl_2_.^146^ Several parameters, including substrate concentrations and the presence of a proton donor (Bu_4_NHSO_4_), were investigated to promote selectivity in the reductions. Under optimized conditions, the authors demonstrated that the titanium­(III) complex, formed by electrochemical reduction of Cp_2_TiCl_2_ (2 equiv), could partially reduce 4,4′-dinitrodibenzyl **16** to 4-amino,4′-nitrodibenzyl **20** at the potential associated with the first reduction wave of Cp_2_TiCl_2_ (−0.80 V vs. SCE) with a selectivity of 9:1 to the fully reduced diamine compound. In contrast, the diamine product **19** was selectively formed when a more negative potential was applied (−1.30 V vs. SCE) (Scheme 4B). It was observed with cyclic voltammetry that the first reduction of Cp_2_TiCl_2_ was not affected by the presence of the dinitrocompound, which led the authors to propose that a homogeneous outer-sphere electron transport is not preferred for reduction of the latter. Instead, the formation of an intermediate complex that involves an electrogenerated Ti­(III) metallocene complex and the dinitro compound, or its corresponding radical anion, was suggested to occur, with subsequent reduction of the latter via an inner-sphere process (Scheme 4C). Interestingly, the addition of the dinitro compound in the absence of the proton donor Bu_4_NHSO_4_ did not result in any significant increase in the cathodic current for the Ti­(IV)/Ti­(III) redox couple, which led the authors to conclude that the Ti-mediated process is inefficient under such conditions. Overall, the process was found to be sluggish, with 50 percent of the initial material recovered after 24 h when 2 equiv of Ti was used. Full conversion could be achieved with a higher load of the Ti-mediator (6 equiv) but resulted in an unselective process with both mono- and direduced products being obtained.

**Scheme 4:**
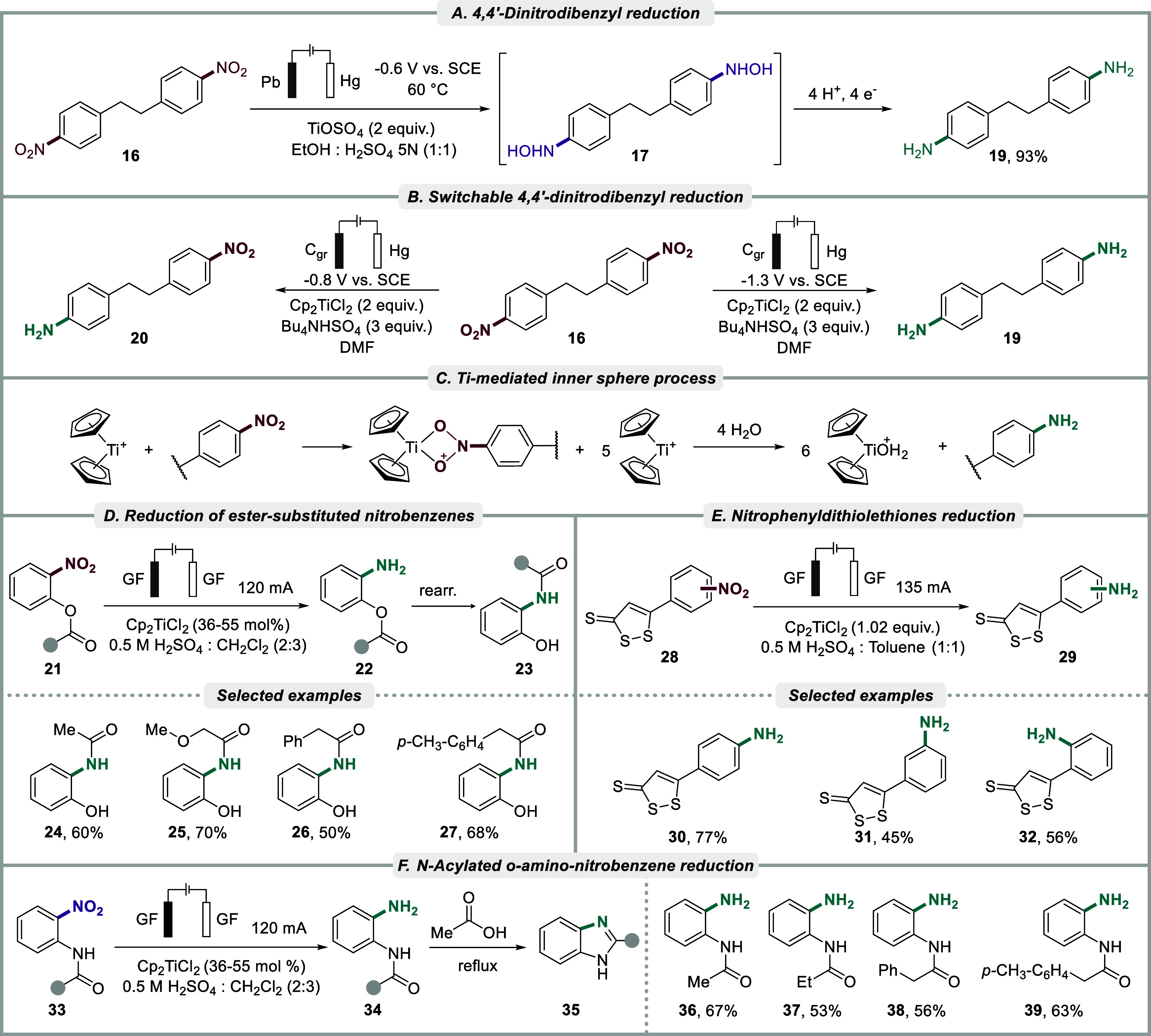
(A) Ti-mediated reduction of 4,4′-dinitrodibenzyl, (B) Selective reduction of 4,4′-dinitrodibenzyl by varying electrode potential, (C) Proposed inner-sphere process of nitro group reduction involving a Ti­(III) metallocene complex, (D) Reduction of *o*-nitrophenyl esters with graphite felt (GF) with rearrangement, (E) Reduction of nitrophenyl-dithiolethiones, and (F) Reduction of N-acetylated *o*-amino-nitrobenzene with formation of imidazoles

Moinet et al. studied the indirect reduction of various nitrobenzene compounds to the corresponding anilines (up to 83% isolated yield) in dichloromethane or toluene using (C_2_H_5_)_2_Ti^+^ as catalyst, electrogenerated from (C_2_H_5_)_2_TiOH^+^ that, in turn, formed via hydrolysis of titanocene dichloride in aqueous acidic medium (1–2 N H_2_SO_4_) under phase transfer conditions.^132^ To prevent degradation of the mediator, the studies were conducted in the dark and under a nitrogen atmosphere using an electrochemical flow cell. The authors used this approach for the reduction of several ortho-substituted nitrobenzenes containing ester (−OCOR), carbonate (−OCO_2_R), amide (−NHCOR) or carbamate (−NHCO_2_R) groups.^133^ In the former case, the formed *o*-aminophenylesters **22** were found to undergo a rearrangement *in situ* to the corresponding *N*-acylated *o*-aminophenols **23** in yields between 50 and 70% (Scheme 4D). For the electroreduction of *o*-nitrophenylamides **33** to N-acylated *o*-aminoanilines **34**, the products were formed in similar yields and were found to be stable under the reaction conditions with the exception of *N*-acetyl *o*-phenylenediamine **36**, which partially cyclized to the corresponding benzimidazole **35**. Upon thermal treatment in acetic acid, all *N*-acylated *o*-aminoanilines were quantitatively transformed into the corresponding benzimidazoles **35** (Scheme 4F). The strategy was successfully extended to encompass the mediated electroreduction of 5-(*o*-, *m*-, and *p*-nitrophenyl)-1,2-dithiole-3-thiones **30** in flow with yields up to 77% (Scheme 4E),^134^ despite the known lability of the 1,2-dithiole-3-thione disulfide bond under reductive conditions.^147,148^ In contrast, direct electrolysis in the absence of the Ti mediator resulted in the irreversible reduction of the dithiolethione substituent.

*N*-Hydroxylamines are commonly considered intermediates in nitro-group electroreduction, but they have also been used as a substrate class in reductive electrosynthetic transformations.^149^ One of the earliest examples was reported by Lund and Feroci, as they examined the electrochemical reduction of hydroxylamine derivatives, including mono and disubstituted *N*-hydroxylamines, aliphatic amine oxides, and heteroaromatic *N*-oxides in the presence of Ti- and Fe-mediators.^150^ While it was demonstrated that the reduction could occur in both acidic, nearly neutral, and alkaline solutions, the reaction rate was found to decrease with the pH for various hydroxylamines. Under optimal conditions, the authors demonstrated that the reduction of 7-(1,3-benzodioxol-5-yl)-2,3,3a,6,7,7a-hexahydro-1-hydroxy-1*H*-indole **40** in a 0.2 M solution of oxalic acid in the presence of an electrogenerated Ti­(III)-mediator (70 mol %), formed from TiCl_4_, resulted in 75% of the amine product **41** (Scheme 5A). Under similar electrochemical conditions, *N*-hydroxypiperidine **42** was reduced to piperidine **44** in 82% yield, supposedly via nitrosamine intermediate **43** (Scheme 5B).

**Scheme 5:**
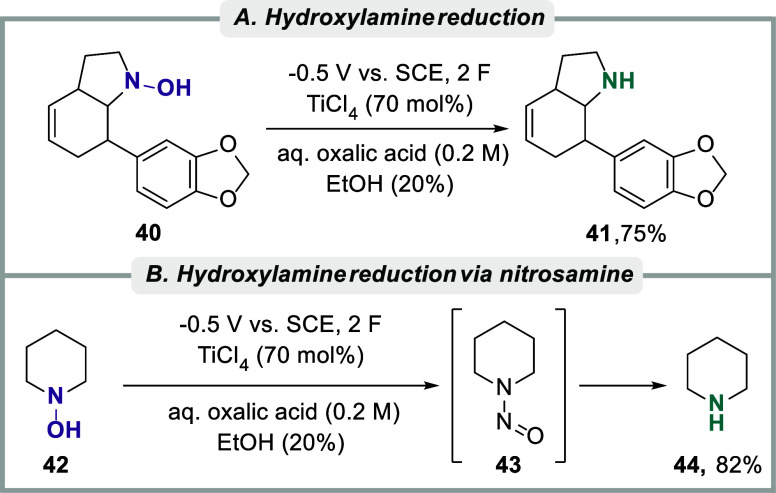
(A) Indirect electrolytic reduction of a hydroxylamine derivative and (B) Hydroxylamine reduction via nitrosamine

Lisitsyn and co-workers studied the electrochemical amination of arenes with hydroxylamine in sulfuric acid in the presence of Ti­(IV) sulfate, leading to the formation of aniline **2** and isomeric phenylenediamines under acidic conditions (Scheme 6A).^151,152^ By examination of the influence of acetic acid on the efficiency and mechanism of substitution in aqueous acidic media, it was proposed that the electrochemically formed Ti­(III) mediator reduces hydroxylamine to the unprotonated radical amine intermediate [Ti­(IV)­NH_2_]^•^, which interacts with the arene to form aniline after displacement of a new molecule of hydroxylamine. In turn, the Ti­(IV) radical amine intermediate ([Ti­(IV)­NH_2_]^•^) can furnish radical cation amines (NH_3_^•^^+^), which can combine with benzene **45** to produce aniline **2**. By introducing acetic acid to the reaction mixture, both reaction rate and selectivity between mono- and diamination of benzene could be tuned. Without the additive, the formation of the disubstituted *p*-phenylenediamine was predominant. In contrast, the monosubstituted product was favored using 2–7 M acetic acid, whereas the yield drastically dropped at higher concentrations (above 9 M). It was hypothesized that the presence of acetic acid modifies the coordination sphere of Ti­(IV) ions, thereby affecting the rearomatization stage of the intermediates. Successive work by the same group extended the Ti-mediated electrolytic amination studies in acidic aqueous/organic media.^153–157^ In this case, it was found that an increase in the concentration of either acetic acid or acetonitrile in the sulfuric acid-containing catholyte significantly speeds up the rate of aniline formation. Similarly, it was found that the current efficiency of isomeric phenylenediamines depends on the acetic acid or acetonitrile concentration. As may be expected based on classic reactivity patterns for electrophilic aromatic substitution, an increase in acid concentration increased the yield for monoaminated product and increased the selectivity for the meta-isomer of the formed dianiline.^153^ Interestingly, the same trend was observed as the concentration of acetonitrile increased, although to a lesser extent. Consistent with these findings, optimized conditions enabled selective monoamination amination of benzene **45** to aniline **2** could be carried out with 79% yield as calculated on hydroxylamine content, using a platinum cathode and a current density of 2 mA/cm^2^ (Scheme 6B).^158^ Similarly, monoamination of anisole with hydroxylamine to furnish *ortho-* and *para-*anisidines was achieved using a similar approach.^159–161^ Furthermore, the selective synthesis of benzene-1,3-diamines **48** by functionalizing *p*-anisidine **46** and *p*-chloroaniline **47** in sulfuric acid solution was reported with yields up to 99% (Scheme 6C).^162,163^ The mechanism for amination of chlorobenzene and chloroanilines in an acidic electrolyte was the topic of separate studies,^164,165^ indicating that the transformation proceeds via *in situ* formation of an amino cation–radical from the hydroxylamine that is attacked by the aromatic nucleophile in an electrophilic aromatic substitution-type mechanism. Along similar lines, Takata and Chiba reported an electrochemical Ti-mediated protocol for amination of conjugated dienes, using hydroxylamine as amine source.^166^ However, in this case, the authors propose that an aminyl radical, formed via reduction of the hydroxylamine by the electrochemically (re)­generated Ti­(III) mediator, in a similar way as in Scheme 5B, attacks 1,3-butadiene **49** to furnish a carbon-centered radical intermediate that undergoes dimerization to linear **50** and branched products **51** (Scheme 6D).

**Scheme 6:**
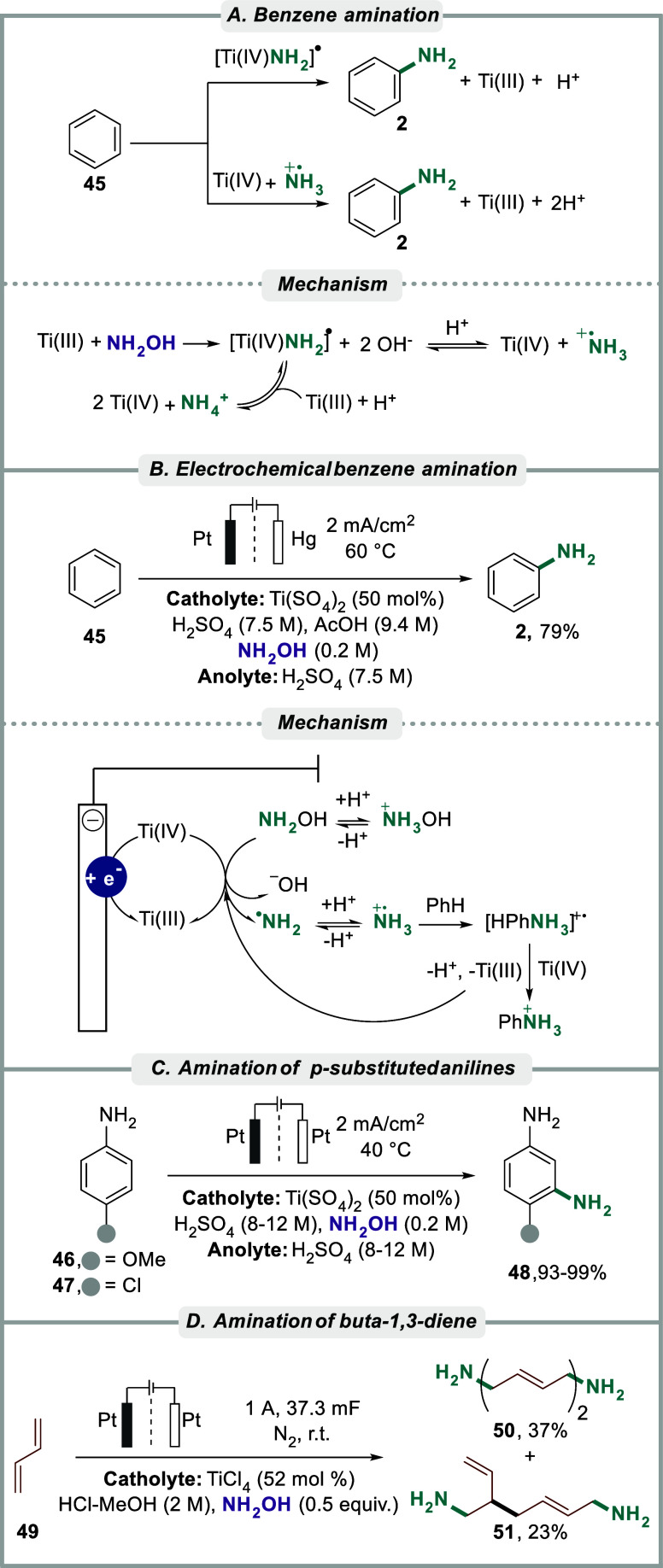
(A) Benzene amination with proposed mechanistic pathways, (B) Electrochemical benzene amination with suggested mechanism, (C) Amination of anilines to diamines, and (D) Electrochemical amination of buta-1,3-diene

## C–X Bond Cleavage

Halogenated organic compounds are essential building blocks in organic synthesis for both fine and bulk chemicals. In a radical manifold, alkyl halides serve as a common source of C-centered alkyl radicals upon single-electron reduction in a stepwise or concerted mechanism and have been utilized for a number of radical cross-electrophile coupling reactions in recent years under chemical,^82,167–173^ photochemical^174–182^ and electrochemical conditions.^92–97,183^ In the context of electrochemically driven titanium-catalysis, Magdesieva et al. studied the reductive dehalogenation of a variety of benzylic halides to the corresponding toluene derivatives using 10–30 mol % Cp_2_TiCl_2_ as precatalyst under potentiostatic electrolysis.^184,185^ These conditions were proposed to electrochemically generate the Nugent–RajanBabu complex, the reactivity of which can be attributed to its unpaired d^1^ electron in combination with its Lewis acidic character that enables it to coordinate Lewis basic sites in organic compounds and induce single-electron transfer via inner-sphere mechanisms.^186–188^ Effectively, this can enable reductive electron transfers to the latter even if its reduction potential has been determined to be more negative than that of the metal complex.^10,109,189^ For Magdesieva et al., electroreduction of benzyl bromide and benzyl chloride in the presence of the Ti-complex resulted in a moderate yield of the hydrodefluorinated products (<30%) after 2 F, electron-withdrawing groups such as nitro in *para*-position **52** improved the efficiency of the transformation, leading to yields up to 75%. In addition to the C–Br and C–Cl bonds, the applied conditions were successfully used for the cleavage of a benzylic C–SCN bond. Notably, the Ti-mediated reduction of benzyl chloride was carried out at more than 1 V more anodic potential compared with that determined for the substrate in the absence of the Ti complex. This catalyst-enabled anodic shift for substrate reduction is interesting from a synthetic point of view as well as from the perspective of energy consumption of the process. The magnitude of the observed anodic shift with CV data and simulations, prompted the authors to postulate an inner-sphere mechanism for the electron transfer from catalyst to the benzyl halide.^10^ In contrast, the nitro-substituted benzyl halides (Scheme 7A) were proposed to undergo intramolecular dissociative electron transfer (DET),^190,191^ with the π* orbital of the nitro group serving as a “redox antenna” that subsequently transferred the accepted electron to the σ* of the C–X bond to afford bond cleavage. Aryl halides did not undergo reductive dehalogenation under the applied conditions. However, the π-activated C–Br bonds in α-bromoketones **54** could be cleaved under similar Ti-catalyzed electroreductive conditions at mild potentials in 38–82% yield using 30–43 mol % of Ti­(Cp)_2_Cl_2_ (Scheme 7B), they also managed to obtain acetophenone in 54% from phenacyl bromide.^192^ Based on the significant difference in reduction potential of nearly 1 V for the benchmark substrate α-bromoacetophenone in the absence and presence of the Ti-catalyst, the authors proposed an inner-sphere radical mechanism for the dehalogenation (Scheme 7C), although the formation of organo-titanium intermediates could not be ruled out.^193,194^

**Scheme 7:**
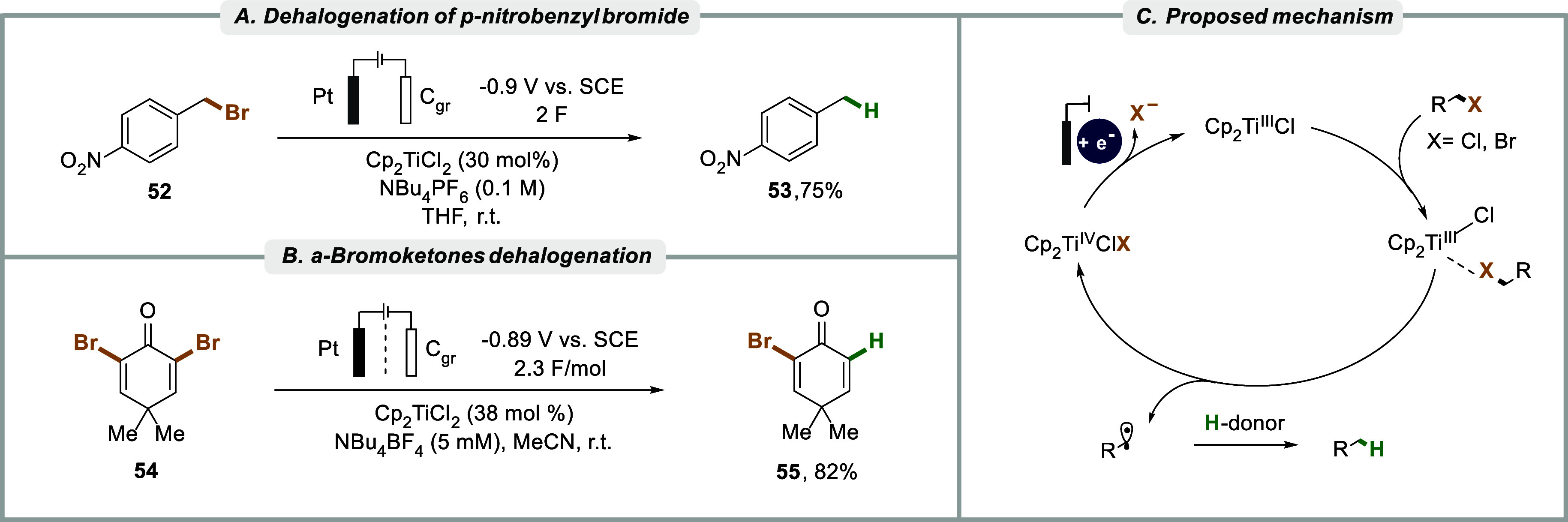
(A) Dehalogenation of *p*-nitrophenyl benzyl bromide, (B) Reductive dehalogenation α-bromoketones, and (C) Proposed mechanism

## C–O Bond Cleavage

The Lewis acidity of Ti-complexes make them good at coordinating oxygen-containing compounds, thereby enabling inner-sphere single-electron transfer from low-valent catalyst states to induce reduction of substrates that would otherwise require more forcing conditions.^11,18,19^ Such behavior was observed by Cosoveanu and co-workers who utilized Cp_2_TiCl_2_ as precatalyst to reduce 9-fluorenone **56** in DMF under potentiostatic conditions, to furnish either the corresponding alcohol **58** or the pinacol product **57** depending on the applied potential (Scheme 8). The selectivity for **58** increased in the presence of a proton donor, the absence of which resulted in lower conversion and a product ratio of about 3:3:1 (**57**:**58**:**59**) at −1.3 V vs SCE.^146^

**Scheme 8:**
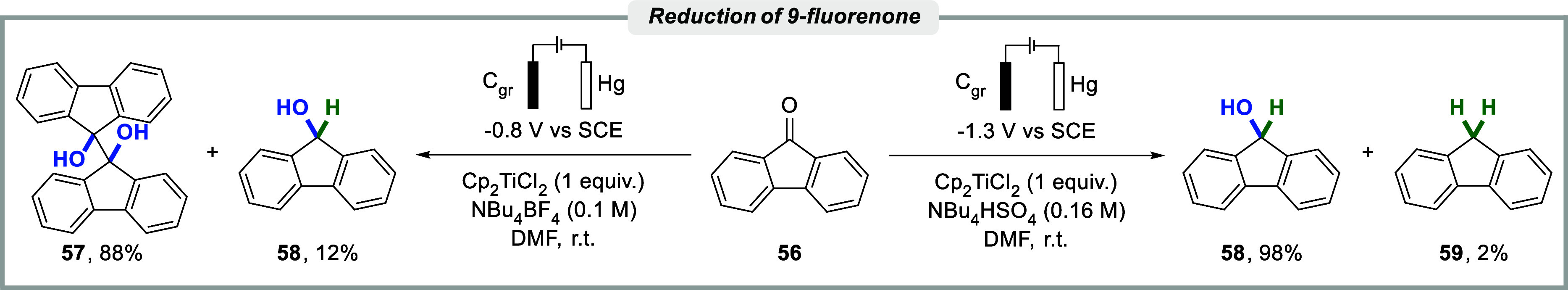
Reduction of 9-fluorenone with product distribution at different potentials

Magdesieva et al. reported on the titanocene-mediated electroreductive ring-opening of epoxides to primary alcohols.^195^ In this case, the authors demonstrated that potentiostatic electrolysis in the presence of Cp_2_TiCl_2_ enabled reductive opening of the oxirane ring in styrene oxide **60** and 4-nitrostyrene oxide at approximately 200 mV more positive potential than that required in the absence of the Ti-catalyst. To prevent direct reduction of the nitro group at the electrode, the electrolysis was conducted at a slightly more anodic value compared to the peak potential for Cp_2_TiCl_2_. The stability of intermediate radical species and steric effects regulate the regioselectivity of this type of reaction, typically resulting in reversed selectivity compared to ring-opening via S_N_2 pathways. Gansäuer and co-workers reported on related Ti­(III)-catalyzed radical epoxide ring-opening reactions, including asymmetric functionalizations with arenes, alkenes, and alkynes, using chemical reductants.^75,196–203^ In an electrochemical setting, the group closely studied titanocene­(III)-catalyzed epoxide ring opening with concomitant intramolecular arylation.^75,197–208^ Based on in-depth studies using cyclic voltammetry, it was found that the efficiency of the catalysis is related to the equilibria between the precatalyst, the resting state of the reduced catalyst [Cp_2_Ti^IV^X_2_]^−^ and the active catalyst Cp_2_TiX (Scheme 9A). To pull the equilibria toward the latter, halide scavengers such as thiourea **63**, squaramide **64** and bis-sulfonamide **65** were successfully employed in THF to promote bulk electrolysis of Cp_2_TiX_2_ (X=Cl, Br, or O_3_SMe) and enable the intramolecular radical epoxide arylation (Scheme 9B).^206^ The electrochemical generation of Ti­(III) species from tetravalent titanocene halides was also reported by Samuel and Vedel.^209^ These monohalide complexes were found to primarily exist as halogen-bridged paramagnetic dimers of the type (Cp_2_Ti^III^X)_2_ (X = Cl, Br, I) in the solid state.^186^ While the dimeric structure was preserved in hydrocarbon solvents, polar coordinating organic solvents commonly used in electrosynthesis were found to favor the formation of a solvated monomer [Cp_2_Ti^III^X­(S)] (S = THF/DMF/pyridine). Electrochemical reduction of titanocenes to the corresponding Ti­(III) species was also studied by Daasbjerg and co-workers by means of cyclic voltammetry and kinetics, displaying a quasi-reversible redox behavior with reversibility decreasing from Cl to Br and I.^189^ Interestingly, electrochemical reduction of Cp_2_TiCl_2_ primarily resulted in the formation of [Cp_2_TiCl_2_]^−^ in THF, whereas the bromide and iodide analogues (X = Br or I) were more prone to form neutral trivalent monomer and dimer complexes Cp_2_TiX and (Cp_2_TiX)_2_, supposedly due to weaker Ti­(III)–X bonds. Notably, formation of such complexes were also promoted from Cp_2_TiCl_2_ when metal reductants such as Zn, Mn, and Al were used, whereas neither [Cp_2_TiCl_2_]^−^ nor Cp_2_Ti^+^ were detected.^193^ In analogy with the findings of Gansäuer, this observation suggests that metal ions may serve the role of halide scavengers in this case. Ligand dissociation was proposed to followed an E_q_C_r_ reaction mechanism (Scheme 9B), as originally proposed by Laviron et al.^210^ Based on kinetic studies using benzyl chloride and benzaldehyde for the formation of dibenzyl and pinacol adducts, respectively, the reactivities of the different Ti­(III) species were found to follow the order (Cp_2_TiCl)_2_ ≥ Cp_2_TiCl ≈ [Cp_2_Ti]^+^ > [Cp_2_TiCl_2_]^−^.

**Scheme 9:**
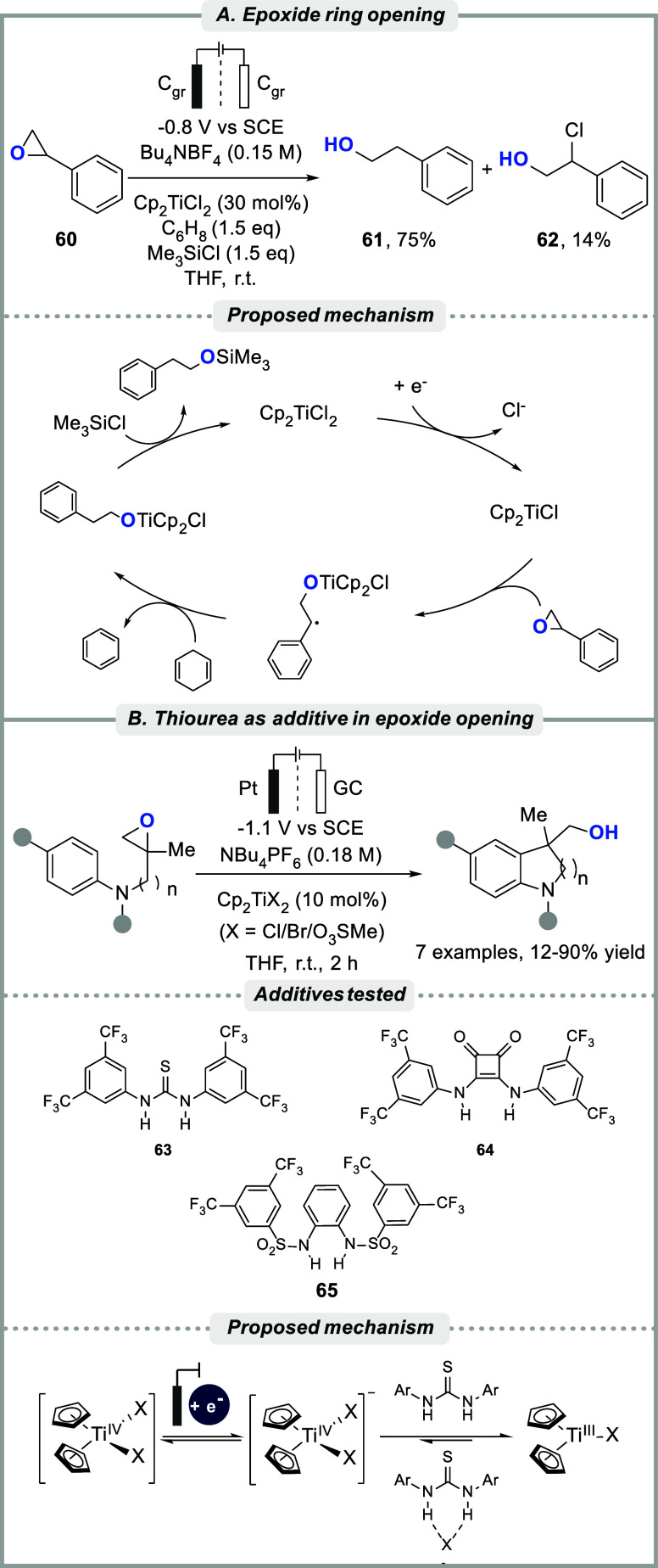
(A) Epoxide opening with proposed mechanism and (B) Epoxide opening with thiourea as halide scavenger

## Conclusion

Titanium catalysis enables a multitude of organic transformations but remains underexplored in the context of electrosynthesis, despite the benefits that electrochemical (re)­generation of catalytically active low-valent species have from the perspectives of sustainability and selectivity. As demonstrated in this Review, the state-of-the-art is limited to N–O bond reductions, reductive dehalogenations, and deoxygenations. Only marginal work has been dedicated to ligand design for well-defined low-valent Ti-complexes for electrochemically driven organic transformations.^211^ Future systematic structure–activity relationships are, thus, likely to result in new mechanistic understanding to drive the development of new transformations, including asymmetric variants.^197–199,201,202^ Furthermore, electrosynthetic conditions may enable the catalytic use of other group IV metals—zirconium and hafnium—in low-valent redox states for catalytic purposes, a field with great untapped potential.^212^ Considering the rich literature on group IV metal-containing materials, such as metal–organic frameworks as well as electrode materials for energy storage applications, it may be envisioned that engineered electrodes with heterogeneous electrocatalyst layers can provide further synthetic opportunities ahead.^213,214^

For several examples shown in this Review, it was demonstrated that the Ti-mediated reductive transformations occurred at more anodic potentials compared to what the redox potential of the substrate alone would dictate. This anodic shift clearly demonstrates the ability of Lewis acidic low-valent Ti complexes to drive challenging electron transfers via inner-sphere mechanisms, which may enable more selective transformations with greater functional group tolerance. In addition, this catalytic ability may be suitable to exploit for energy- and waste-efficient remediation of halogenated chemical waste-streams, including chlorinated polymers and pesticides, as well as for biomass valorization purposes. With its stability in different redox states, its high abundance, and low price and toxicity, titanium holds great promise for application in future electrocatalytic transformations of organic compounds.
